# Discovering Relations Between Mind, Brain, and Mental Disorders Using Topic Mapping

**DOI:** 10.1371/journal.pcbi.1002707

**Published:** 2012-10-11

**Authors:** Russell A. Poldrack, Jeanette A. Mumford, Tom Schonberg, Donald Kalar, Bishal Barman, Tal Yarkoni

**Affiliations:** 1 Imaging Research Center and Departments of Psychology and Neurobiology, University of Texas, Austin, Texas, United States of America; 2 NASA Ames Research Center, Mountain View, California, United States of America; 3 Department of Electrical and Computer Engineering, University of Texas, Austin, Texas, United States of America; 4 Department of Psychology, Colorado University, Boulder, Colorado, United States of America; Indiana University, United States of America

## Abstract

Neuroimaging research has largely focused on the identification of associations between brain activation and specific mental functions. Here we show that data mining techniques applied to a large database of neuroimaging results can be used to identify the conceptual structure of mental functions and their mapping to brain systems. This analysis confirms many current ideas regarding the neural organization of cognition, but also provides some new insights into the roles of particular brain systems in mental function. We further show that the same methods can be used to identify the relations between mental disorders. Finally, we show that these two approaches can be combined to empirically identify novel relations between mental disorders and mental functions via their common involvement of particular brain networks. This approach has the potential to discover novel endophenotypes for neuropsychiatric disorders and to better characterize the structure of these disorders and the relations between them.

## Author Summary

One of the major challenges of neuroscience research is to integrate the results of the large number of published research studies in order to better understand how psychological functions are mapped onto brain systems. In this research, we take advantage of a large database of neuroimaging studies, along with text mining methods, to extract information about the topics that are found in the brain imaging literature and their mapping onto reported brain activation data. We also show that this method can be used to identify new relations between psychological functions and mental disorders, through their shared brain activity patterns. This work provides a new way to discover the underlying structure that relates brain function and mental processes.

## Introduction

The search for clues regarding the underlying causes of mental disorders has led to the notion that these disorders may be best understood in terms of a set of underlying psychological and/or neural mechanisms that stand between genes and environment on the one hand and psychiatric diagnoses on the other hand. Such intermediate phenotypes, or “endophenotypes”, may provide the traction that has eluded research using diagnostic categories as primary phenotypes [1], [2]. They may also provide the means to better understand the structure the underlying psychological dimensions that appear to underlie overlapping categories of mental disorders [3], [4].

The identification of endophenotypes requires an understanding the basic structure of mental functions and their associated brain networks. For more than 30 years, cognitive neuroscientists have used neuroimaging methods (including EEG/MEG, PET, and fMRI) in an attempt to address this question. This work has led to a large body of knowledge about associations between specific psychological processes or tasks and activity in brain regions or networks. However, this knowledge has not led to a commensurate improvement in our understanding of the basic mental operations that may be subserved by particular brain systems. Instead, diverse literatures often assign widely varying functions to the same networks. A prime example is the anterior cingulate cortex, which has been associated with such widespread functions as conflict monitoring, error processing, pain, and interoceptive awareness. In order to understand the unique functions that are subserved by brain regions or networks, a different approach is necessary; namely, we need to analyze data obtained across a broad range of mental domains and understand how these domains are organized with regard to neural function and structure.

The identification of basic operations can be understood statistically as a problem of latent structure identification; that is, what are the latent underlying mental functions and brain networks that give rise to to the broad range of observed behaviors and patterns of brain activity and neuropsychiatric disorders? The focus within cognitive neuroscience on establishing associations between activation and specific hypothesized processes has hindered the ability to identify such latent structures. However, within the fields of machine learning and text mining, a number of powerful approaches have been developed to estimate the latent structure that generates observed data, assuming that large enough datasets are available. In the present work, we take advantage of one class of such generative models to develop a new approach to identifying the underlying latent structure of mental processing and the associated brain functions, which we refer to as “topic mapping”. We examine the latent conceptual structure of the fMRI literature by mining the full text from a large text corpus comprising more than 5,800 articles from the neuroimaging literature, and model the relation between these topics and associated brain activation using automated methods for extracting activation coordinates from published papers. This analysis uncovers conceptual structure and activation patterns consistent with those observed in previous neuroimaging meta-analyses, which provides confirmation of the approach, while also providing some novel suggestions regarding structure/function relationships. We then use this approach to identify the topical structure of terms related neuropsychiatric diseases, and use multivariate methods to identify relations between these the mental and disorder domains based on common brain activation patterns. This approach provides an empirical means of discovering novel endophenotypes that may underlie mental disorders, as well providing new insights into the relations between diagnostic categories.

Within the fields of information retrieval and computer science, research into document retrieval has led to the development of a set of techniques for estimating the latent structure underlying a set of documents. Early work in this area treated documents as vectors in a high-dimensional space, and used matrix decomposition techniques such as singular value decomposition to identify the latent semantic structure of the documents [5]. More recently, researchers in this domain have developed approaches that are based on generative models of documents. One popular approach, known generically as “topic models” [6], treats each document as a mixture of a small number of underlying “topics”, each of which is associated with a distribution over words. Generating a document via this model involves sampling a topic and then sampling over words within the chosen topic; using Bayesian estimation techniques, it is possible to invert this model and estimate the topic and word distributions given a set of documents. The particular topic modeling technique that we employ here, known as latent Dirichlet allocation (LDA: [7]), has been shown to be highly effective at extracting the structure of large text corpuses. For example [8], used this approach to characterize the topical structure of science by analyzing 10 years of abstracts from *PNAS*, showing that it was able to accurately extract the conceptual structure of this domain.

## Results

**Figure 1:**
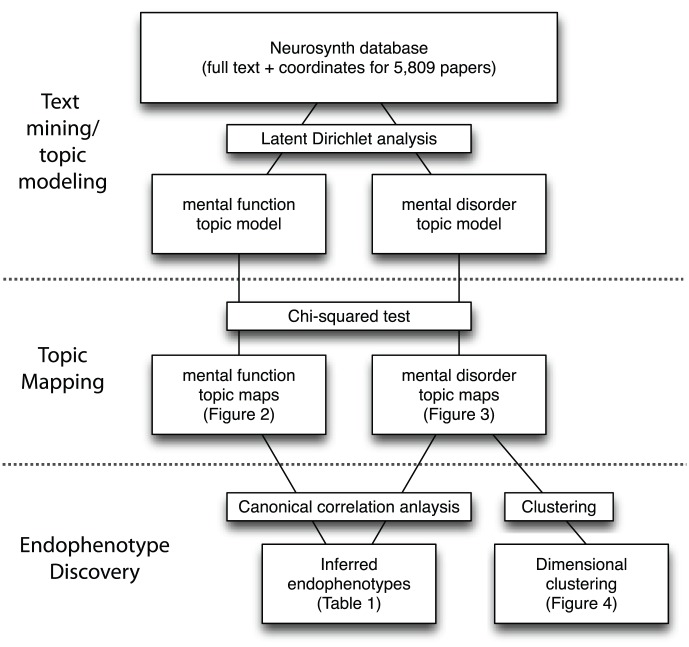
A schematic overview of the data processing pipeline used in the analyses presented here.

**Figure 2:**
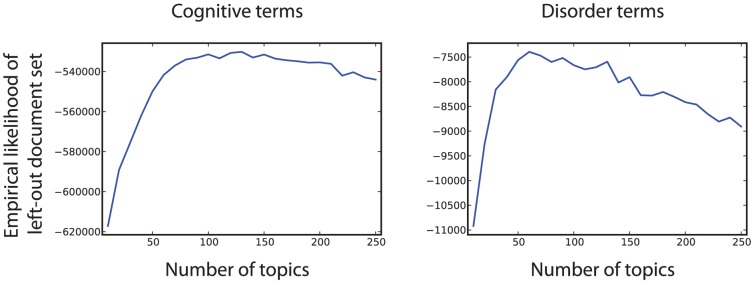
Plots of the average empirical likelihood of the left-out document sets across cross validation folds, for cognitive terms (left) and disorder terms (right).

**Figure 3:**
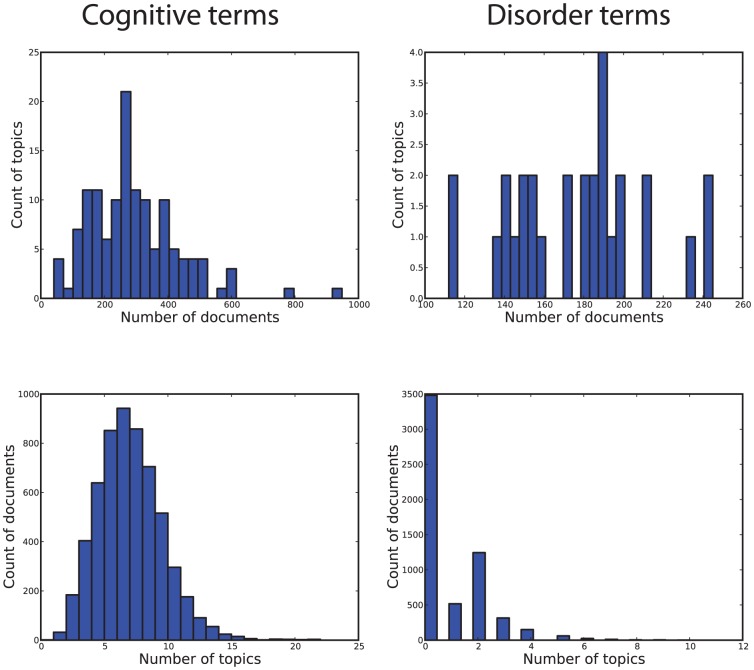
Histograms of the number of topics per document (top row) and documents per topic (bottom row) for cognitive terms (left column) and disorder terms (right column).

We characterized the latent structure of the cognitive neuroscience literature by applying latent Dirichlet allocation to a corpus of 5,809 articles (using an expanded version of the corpus developed in [9]), which were selected on the basis of reporting fMRI activation in a standardized coordinate format. An overview of the entire data processing workflow is presented in Figure 1. This technique estimates a number of underlying latent “topics” that generate the observed text, where each topic is defined by a distribution over words. The dimensionality (i.e., number of topics) is estimated using a cross-validation approach; the documents are randomly split into 8 sets, and for each set a topic model is trained on the remaining data and then used to estimate the empirical likelihood of the held-out documents [10]. Plots of the empirical likelihood of left-out documents as a function of the number of topics are shown in Figure 2, and histograms of the number of documents per topic and number of topics per document are shown in figure 3.

**Figure 4:**
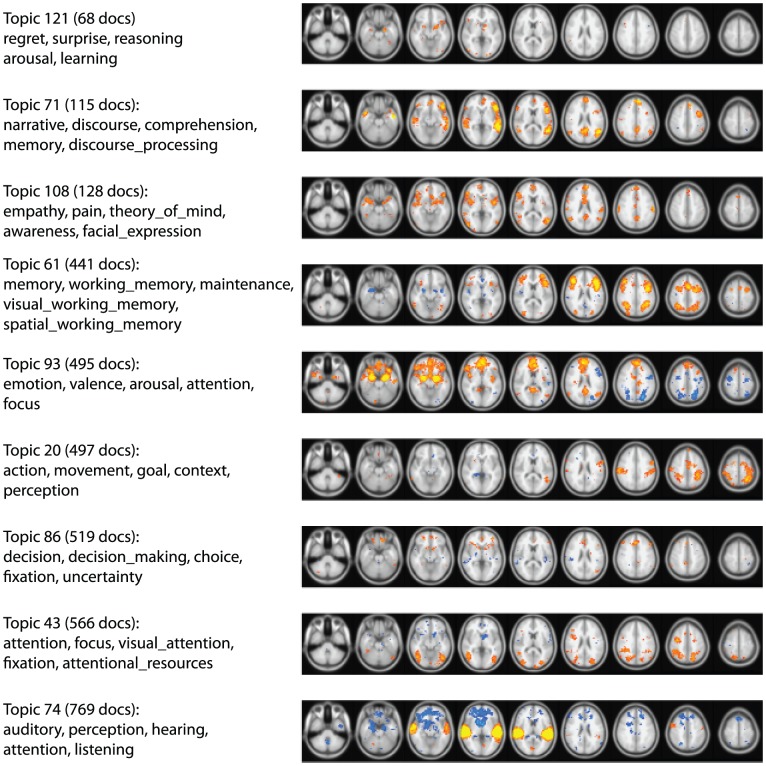
Examples of mental function topics and associated topic maps. The left panel shows the top words associated with each topic, and the right panel shows a map of voxels that were significantly associated with loading on that topic across documents. The image intensity is proportional to the Pearson correlation between the activation vector and the topic loading vector at each voxel (with red-yellow depicting positive correlations and blue-white depicting negative correlations), thresholded using a whole brain false discovery rate of q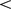.01. The topics are shown in order of descending number of documents with nonzero loadings on the topic; those at the top showed loading on a relatively small number of documents, whereas those at the bottom showed loading across a broader set of documents. The images are presented in radiological convention (i.e., left-right reversed).

Initial application of LDA to the full-text corpus identified a number of topics that were related to mental function, but also many topics related to methodological or linguistic aspects of the documents. Because we were specifically interested in estimating the conceptual structure of mental processes, we examined each document in the corpus and identified each occurrence of any of the 605 terms (both single words and phrases) that are present as mental concepts in the Cognitive Atlas (http://www.cognitiveatlas.org); the topic model was then estimated using this limited word set (treating each word or phrase as a single-word token). The Cognitive Atlas is a curated collaborative ontology that aims to describe mental functions, and contains terms spanning across nearly all domains of psychological function [11]. The cross-validation analysis identified 130 as the optimal number of topics for this dataset. Examples of these topics are shown in Figure 4, and the full list is presented in Table S1. In large part these topics are consistent with the topics that are the focus of research in the cognitive neuroscience literature. The topics with the highest number of associated documents were those related to very common features of neuroimaging tasks such as movement (topic 20), emotion (topic 93), audition (topic 74), attention (topic 43), and working memory (topic 61). Each of these was associated with more than 400 documents in the corpus. At the other end of the spectrum were more focused topics that loaded on fewer than 200 documents, such as topic 121 (regret,surprise), topic 71 (narrative, discourse), and topic 108 (empathy, pain). The results of this analysis suggest that topic modeling applied to the limited term set of mental functions can successfully extract the conceptual structure of psychological processes at multiple levels within the current text corpus.

**Figure 5:**
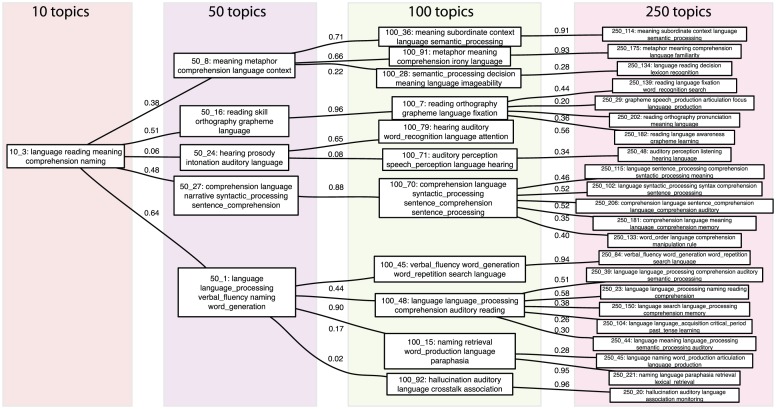
A hierarchical graph depicting topics involving the term “language” across multiple topic dimensionalities. All topics with “language in their top 5 terms were first identified from the results for topic models fit to the data at 10, 50, 100, and 250 topics. At each level, each topic is linked to the topic at the previous level with which it had the highest correlation in its document loadings. The values on each edge reflect the correlation in the topic loading vector across documents between the two levels.

In order to further examine the effects of topic dimensionality, we compared the results obtained across several values for the number of topics (10,50, 100, and 250). We chose the term “language” and identified all topics for each model in which that term occurred in the top five terms. We then examined the correlation in the loading vector across documents for each set of levels, in order to identify the hierarchical graph relating topics across levels (see Figure 5). This analysis showed that increasing the topic dimensionality resulted in finer-grained topics; for example, with 10 topics there was a single matching topic that included “meaning”, “reading”, and “comprehension”, whereas each of these was split into a separate set of topics in the 50-topic model, and further subdivided as the dimensionality increased. This suggests that although the cross validation resulted in a particular “best” dimensionality, in reality there is relevant information at many different levels which differs in grain size.

### Topic mapping

Using the topical structure of the literature discovered in the previous section, we developed a novel approach called *topic mapping* in which we identify the relationship between brain activation and topic loading in order to characterize the neural systems associated with these topics. The distribution of topics across documents was used to identify the neural substrates of each topic across all of the studies in the corpus. For each paper in the database, the reported activation coordinates were obtained from Neurosynth (http://www.neurosynth.org), a database of coordinates automatically extracted from 5,809 articles [9]. Neural activation for each study was then reconstructed by placing a 10 mm sphere at each activation coordinate reported in the paper. This resulted in a binary reconstructed activation map. The document-topic distribution obtained from the topic model for each topic was binarized and used to perform a chi-squared test measuring the association between topic loading and brain activation at each voxel in the brain. Voxels were excluded if the minimum expected frequency under independence was less than 5. Correction for multiple tests across voxels was performed using the voxelwise false discovery rate correction (q
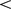
0.01) [12] on the p-values obtained from the chi-squared test.

Figure 4 shows examples of topic maps obtained from this analysis using the 130 topics obtained from the Cognitive Atlas topic model. These maps are largely concordant with known functional neuroanatomy. For example, topic 43 (with terms related to visual attention) was associated with activity in the bilateral lateral occipital cortex, parietal cortex, and frontal cortex. Topic 86 (with terms related to decision making and choice) was associated with regions in the ventral striatum, medial, orbital, and dorsolateral prefrontal cortex. Topic 93 (with terms related to emotion) was associated with bilateral activity in the amygdala, orbitofrontal cortex, and medial prefrontal cortex. These results highlight the fact that this unsupervised approach obtains results that are consistent with the known literature. The topics varied substantially in the extent of significant association; although this may in some cases reflect lower power for topics that are associated with a smaller number of documents, in many cases topics with similar numbers of documents showed very different degrees of association (e.g., topic 86 vs. 93). It should be noted that in all cases the associations were very strong, with p-values usually below p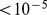. Thus, differences between these maps likely reflect real differences in the extent of activation observed across the literature for these different concepts.

While concordance with the existing literature is reassuring, the true promise of this approach is in its ability to uncover novel associations between functions and activation, and the topic mapping analysis did in fact identify some unexpected associations, particularly when looking at negative associations. Two interesting examples are evident in Figure 4. First, topic 61 was associated with the bilateral fronto-parietal network usually associated with working memory, but it also exhibited strong and focused negative association in the right amygdala; this means that the amygdala was significantly less likely to be activated in studies that loaded on this topic relative to those that did not. This is particularly interesting in light of further exploration of the literature using the PubBrain tool (http://www.pubbrain.org) which identified a number of studies that have noted amygdala activation in association with working memory tasks (cf. [13]). Another example is topic 71 (associated with auditory processing) which was negatively associated with activation in a broad set of regions previously implicated in emotional function, such as orbitofrontal cortex, striatum, and amygdala. Whether such negative associations reflect truly negative relations in activation between these networks or reflect features of the tasks used in these domains remains to be determined, but such unexpected associations could suggest novel hypotheses about relations between specific brain networks. These are only two examples of potential novel discoveries using Topic Mapping.; future studies will be needed to systematically examine all possible new findings emerging from the usage of this tool.

### Mapping the neural basis of neuropsychiatric disorders

**Figure 6:**
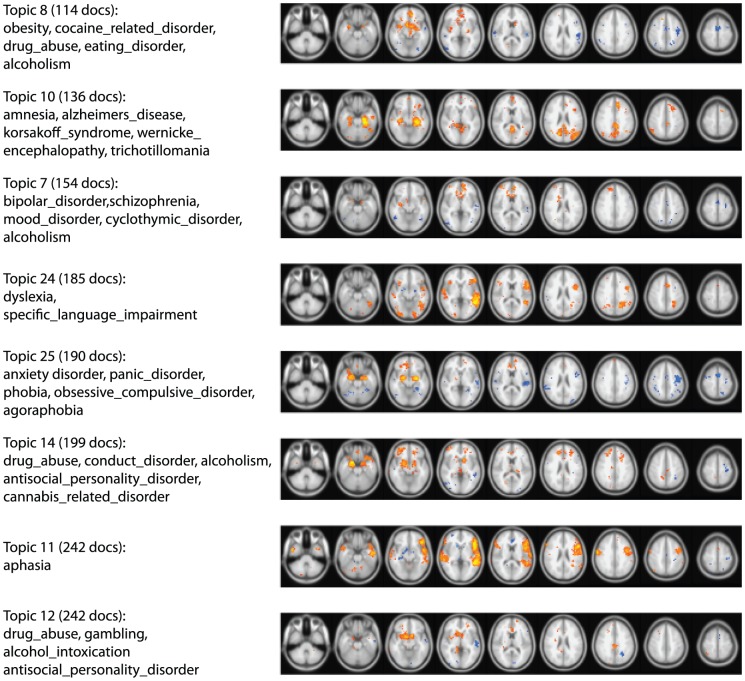
Examples of topic maps based on a topic model limited to disorder-related terms. Topics are ordered in terms of the number of documents loading on the topic; color maps reflect the correlation coefficient between topic loading and activation across documents. The images are presented in radiological convention (i.e., left-right reversed).

Based on the results from the foregoing analyses, we then examined whether it was possible to obtain new insights about the organization of brain disorders using the topic mapping approach developed above. We estimated a set of topics using only terms related to brain disorders, based on a lexicon of mental disorders terms derived from the NIFSTD Dysfunction ontology [14] along with the DSM-IV. The optimal dimensionality of 60 based on cross-validation was found to produce multiple topics with exactly the same word distribution, so we used the largest number of topics yielding a unique set of word distributions across topics, which was 29 topics. Examples of these topics and the associated topic maps are presented in Figure 6.

The results of this analysis are largely consistent with results from prior meta-analyses and known functional anatomy of the various disorders, but are novel in highlighting relations between some of the disorders. For example, Topic 7 demonstrates the relations between bipolar disorder, schizophrenia, and mood disorders, with activation centered on the medial prefrontal cortex, basal ganglia, and amygdala. Topic 8 highlights relations between obesity and eating disorders and drug abuse, with activation in the ventral striatum and ventromedial prefrontal cortex. Topic 14 demonstrates relations between a set of externalizing disorders (drug abuse, conduct disorder, alcoholism, antisocial personality disorder, and cannabis related disorder) with activation focused in the striatum, amygdala, orbitofrontal cortex, and dorsal prefrontal cortex. Conversely, Topic 25 demonstrates relations between a set of internalizing disorders (anxiety disorder, panic disorder, phobia, obsessive compulsive disorder, agoraphobia, and post traumatic stress disorder), with a very similar pattern of activation, though notably weaker in the striatum. One striking result of these analyses is the similarity of the patterns of brain activity associated with the mention of all of these different disorders. This could arise either from the fact that this particular set of limbic brain systems is the seat of all major psychiatric disorders, or the fact that these disorders are commonly mentioned in relation to tasks or cognitive domains that happen to preferentially engage these brain systems.

**Figure 7:**
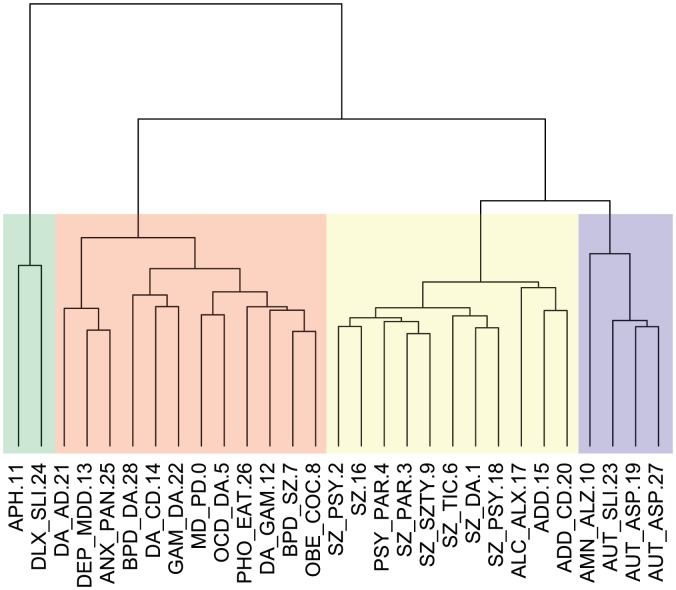
A clustering denodrogram showing the relationships between the different disorder topics based on their distance in neural activation space. Euclidean distance was used as the distance metric for clustering, and hierarchical clustering was performed using Ward's method. The colored blocks show the four major groupings obtained by cutting the tree at a height of 2.0. Abbreviations: APH: aphasia, DLX:dyslexia, SLI: specific language impairment, DA: drug abuse, AD:Alzheimer's disease, DEP:depressive disorder, MDD:major depressive disorder, ANX:anxiety disorder, PAN: panic disorder, BPD: bipolar disorder, CD: conduct disorder, GAM: gambling, MD: mood disorder, PD: Parkinson's disease, OCD: obsessive compulsive disorder, PHO: phobia, EAT: eating disorder, SZ: schizophrenia, OBE: obesity, COC: cocaine related disorder, PSY: psychotic disorder, PAR: paranoid disorder, SZTY: schizotypal personality disorder, TIC: tic disorder, ALC: alcoholism, ALX: alexia, ADD: attention deficit disorder, AMN: amnesia, AUT: autism, ASP: Asperger syndrome.

We further characterized the relations between different disorder concepts in their associated neural activations by clustering the disorder topics based on their associated brain activation patterns using hierarchical clustering. The results of this analysis are shown in Figure 7. The results show the degree to which the neural patterns associated with the use of particular sets of mental disorder terms exhibit a consistent systematic structure. The clustering breaks into four large groups, comprising language disorders, mood/anxiety disorders and drug abuse, psychotic disorders, and autism and memory disorders. What is particularly interesting is that, although none of the topic maps associated with the term “schizophrenia” showed strong activation, the fact that they cluster together in this analysis suggests that they are nonetheless similar in the patterns of activation that are reported in the associated papers; however, this could also reflect the fact that a relatively small number of tasks is used in the literature, and thus any concordance could be driven by overlap of tasks that are commonly mentioned in the context of schizophrenia. Despite such limitations, these results provide further confirmation that the present analysis, while largely based on studies involving healthy adults, can nonetheless accurately characterize the neural basis of mental disorders as described in the literature.

### Empirical discovery of endophenotypes

It has commonly been proposed that cognitive functions and neural systems may serve as endophenotypes for neuropsychiatric disorders. We assessed whether it was possible to empirically discover candidate endophenotypes using a multivariate approach to identify sets of mental concepts and disorder terms that were closely associated via their respective activation patterns. We used an 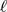
1-penalized non-negative version of canonical correlation analysis (CCA) [15] to identify novel relations between sets of mental functions and sets of neuropsychiatric disorders, based on their associated topic maps. The sparsity penalty was used in order to identify components that are associated with small numbers of topics, in order to improve the interpretability of the resulting canonical variates. This analysis identified 8 sets of disorders and cognitive functions that were related via their associated patterns of activation. The results, shown in Table 1, highlight some interesting relations between the different disorders and psychological functions.

**Table 1:** Canonical variates obtained using sparse canonical correlation analysis on neural activation data for mental concept and disorder topics.

CV #	Mental topics	Disorder topics
0	77 (0.25): mood induction	25 (0.40): anxiety_disorder panic_disorder
	94 (0.25): reward decision	13 (0.36): depressive_disorder major_depressive_disorder
	15 (0.24): reward anticipation	22 (0.35): gambling drug_abuse
	40 (0.23): fear generalization	8 (0.35): obesity cocaine_related_disorder
	105 (0.23): emotion sadness	14 (0.32): drug_abuse conduct_disorder
1	93 (0.24): emotion valence	10 (0.42): amnesia alzheimers_disease
	105 (0.23): emotion sadness	3 (0.39): schizophrenia paranoid_schizophrenia
	39 (0.22): valence arousal	9 (0.37): schizophrenia schizotypal_personality_disorder
	33 (0.22): memory retrieval	23 (0.32): autism specific_language_impairment
	44 (0.21): risk decision	18 (0.30): schizophrenia psychotic_disorder
2	66 (0.27): language syntactic_processing	24 (0.69): dyslexia specific_language_impairment
	13 (0.26): language comprehension	11 (0.68): aphasia
	107 (0.25): language language_processing	27 (0.20): autism asperger_syndrome
	26 (0.25): comprehension language	
	5 (0.25): word_frequency decision	
3	15 (0.29): reward anticipation	0 (0.54): mood_disorder parkinsons_disease
	117 (0.27): anticipation feedback	15 (0.41): attention_deficit_disorder
	94 (0.27): reward decision	20 (0.39): attention_deficit_disorder conduct_disorder
	77 (0.24): mood induction	12 (0.35): drug_abuse gambling
	44 (0.23): risk decision	5 (0.24): obsessive_compulsive_disorder drug_abuse
4	113 (0.30): encoding memory	10 (0.77): amnesia alzheimers_disease
	101 (0.27): recognition memory	17 (0.60): alcoholism alexia
	36 (0.27): memory explicit_memory	
	79 (0.25): familiarity recognition	
	7 (0.25): encoding memory	
5	129 (0.43): cognition social_cognition	19 (0.66): autism asperger_syndrome
	1 (0.34): belief theory_of_mind	27 (0.59): autism asperger_syndrome
	108 (0.30): empathy pain	23 (0.36): autism specific_language_impairment
	45 (0.28): intention prospective_memory	
	71 (0.27): narrative discourse	
11	58 (0.45): emotion facial_expression	26 (0.57): phobia eating_disorder
	49 (0.40): fear emotion	0 (0.44): mood_disorder parkinsons_disease
	99 (0.37): facial_expression emotional_expression	17 (0.42): alcoholism alexia
	40 (0.30): fear generalization	16 (0.35): schizophrenia
	123 (0.28): stress induction	2 (0.30): schizophrenia psychotic_disorder
22	59 (0.41): intelligence morphology	21 (0.70): drug_abuse alzheimers_disease
	88 (0.38): focus attention	4 (0.46): psychotic_disorder paranoid_disorder
	14 (0.31): association context	7 (0.41): bipolar_disorder schizophrenia
	3 (0.29): memory episodic_memory	19 (0.30): autism asperger_syndrome
	35 (0.26): hallucination auditory	

The top five topics for each canonical variate exceeding a loading value of 0.2 are shown in the table.

The first canonical variate (#0) demonstrated associations between a number of both internalizing and externalizing disorders (anxiety, depression, obesity, gambling) which were centered around the involvement of emotional processes (such as mood and fear) and reward-related decision processes. Another canonical variate (#1) was focused on memory processes, and identified a cluster of disorders including classical memory disorders (amnesia and Alzheimer's disease) as well as schizophrenia. Another (#2) focused on language processes and was associated with activity in left prefrontal, temporal, and parietal regions.

The results of the CCA analysis provide a potential new window into the complex psychological and neural underpinnings of schizophrenia and its relation to other psychiatric disorders. Across different canonical variates, schizophrenia is related to mood and decision making processes (components 0 and 3), memory processes (component 5), and social perception (component 10). These could potentially relate to different aspects of schizophrenic symptomatology, such as the distinctions between positive versus negative symptoms or between cognitive versus affective impairments. Further, they provide novel potential targets for genetic association studies, which have struggled to identify meaningful and replicable associations between schizophrenic symptoms or endophenotypes and genetic polymorphisms (cf. [16]).

We also performed CCA directly using topic-document loading vectors, in order to determine whether the results differed from CCA computed on neural loading vectors; the results are presented in Table 2. The results of this analysis are quite concordant with the foregoing analyses based on activation patterns, but one noticeable difference between the two analyses is that the activation-based CCA analysis appeared to cluster disorders more broadly, whereas many of the components found in the text-based analysis had only a single disorder. This may reflect the fact that disorders are less neurally distinct than is suggested by what is written by authors, but could also reflect greater noise in the neural data; further work will be necessary to better understand the unique contributions of activation-based and text-based analyses.

**Table 2:** Canonical variates obtained using sparse canonical correlation analysis directly on document/topic loading distributions for cognitive and disorder topics.

CV #	Mental topics	Disorder topics
0	93 (0.35): emotion valence	22 (0.42): gambling drug_abuse
	77 (0.32): mood induction	13 (0.42): depressive_disorder major_depressive_disorder
	44 (0.31): risk decision	25 (0.41): anxiety_disorder panic_disorder
	94 (0.31): reward decision	26 (0.38): phobia eating_disorder
	49 (0.29): fear emotion	28 (0.23): borderline_personality_disorder drug_abuse
1	62 (0.71): reading language	24 (0.86): dyslexia specific_language_impairment
	72 (0.35): reading language	11 (0.48): aphasia
	107 (0.31): language language_processing	
	5 (0.23): word_frequency decision	
2	129 (0.81): cognition social_cognition	19 (0.66): autism asperger_syndrome
	58 (0.28): emotion facial_expression	27 (0.49): autism asperger_syndrome
	84 (0.20): gaze attention	23 (0.46): autism specific_language_impairment
3	32 (0.47): naming retrieval	11 (1.00): aphasia
	107 (0.44): language language_processing	
	26 (0.32): comprehension language	
	60 (0.32): auditory speech_production	
	66 (0.31): language syntactic_processing	
4	90 (0.69): inhibition response_inhibition	20 (0.69): attention_deficit_disorder conduct_disorder
	11 (0.52): attention sustained_attention	15 (0.66): attention_deficit_disorder
	122 (0.27): attention selective_attention	
	8 (0.21): cognitive_control monitoring	
5	33 (0.76): memory retrieval	10 (1.00): amnesia alzheimers_disease
	3 (0.36): memory episodic_memory	
	64 (0.24): retrieval memory	
6	35 (0.62): hallucination auditory	1 (0.61): schizophrenia drug_abuse
	44 (0.36): risk decision	4 (0.42): psychotic_disorder paranoid_disorder
	17 (0.33): verbal_fluency word_generation	6 (0.36): schizophrenia tic_disorder
	70 (0.31): memory working_memory	16 (0.29): schizophrenia
	14 (0.23): association context	18 (0.27): schizophrenia psychotic_disorder
7	40 (0.71): fear generalization	26 (0.82): phobia eating_disorder
	49 (0.55): fear emotion	25 (0.55): anxiety_disorder panic_disorder
	73 (0.28): arousal attention	
8	77 (0.92): mood induction	13 (0.80): depressive_disorder major_depressive_disorder
	93 (0.22): emotion valence	7 (0.57): bipolar_disorder schizophrenia
9	86 (0.56): decision decision_making	22 (0.98): gambling drug_abuse
	100 (0.41): choice decision	
	94 (0.39): reward decision	
	15 (0.33): reward anticipation	
	44 (0.33): risk decision	
10	98 (0.60): stress association	28 (0.99): borderline_personality_disorder drug_abuse
	67 (0.45): maintenance distraction	
	93 (0.29): emotion valence	
	105 (0.28): emotion sadness	
	81 (0.23): hearing auditory	
11	78 (0.38): movement motor_control	5 (0.97): obsessive_compulsive_disorder drug_abuse
	21 (0.37): interference interference_resolution	0 (0.23): mood_disorder parkinsons_disease
	76 (0.35): planning motor_planning	
	124 (0.34): feedback learning	
	8 (0.27): cognitive_control monitoring	
12	75 (0.59): retention consolidation	9 (1.00): schizophrenia schizotypal_personality_disorder
	26 (0.34): comprehension language	
	96 (0.29): context context_memory	
	1 (0.26): belief theory_of_mind	
	127 (0.23): memory encoding	
13	54 (0.70): desire habit	8 (0.80): obesity cocaine_related_disorder
	15 (0.33): reward anticipation	21 (0.43): drug_abuse alzheimers_disease
	94 (0.31): reward decision	12 (0.35): drug_abuse gambling
	9 (0.28): executive_function attention	
	65 (0.25): recall humor	
14	3 (0.60): memory episodic_memory	17 (1.00): alcoholism alexia
	48 (0.44): metaphor meaning	
	117 (0.25): anticipation feedback	
	62 (0.23): reading language	
	125 (0.22): skill learning	
15	17 (0.50): verbal_fluency word_generation	6 (1.00): schizophrenia tic_disorder
	45 (0.36): intention prospective_memory	
	111 (0.33): memory working_memory	
	97 (0.30): awareness consciousness	
	4 (0.29): cognition recognition	
16	70 (0.57): memory working_memory	16 (0.99): schizophrenia
	96 (0.33): context context_memory	
	58 (0.30): emotion facial_expression	
	10 (0.27): rehearsal memory	
	74 (0.23): auditory perception	
17	4 (0.45): cognition recognition	0 (1.00): mood_disorder parkinsons_disease
	128 (0.39): movement focus	
	51 (0.38): learning sequence_learning	
	38 (0.35): categorization prototype	
	124 (0.29): feedback learning	
18	18 (0.63): lying deception	14 (1.00): drug_abuse conduct_disorder
	64 (0.34): retrieval memory	
	98 (0.30): stress association	
	99 (0.24): facial_expression emotional_expression	
19	84 (0.50): gaze attention	23 (1.00): autism specific_language_impairment
	85 (0.32): inference knowledge	
	108 (0.29): empathy pain	
	45 (0.25): intention prospective_memory	
	4 (0.25): cognition recognition	
20	45 (0.46): intention prospective_memory	3 (1.00): schizophrenia paranoid_schizophrenia
	35 (0.44): hallucination auditory	
	21 (0.41): interference interference_resolution	
	81 (0.27): hearing auditory	
	3 (0.25): memory episodic_memory	
22	8 (0.75): cognitive_control monitoring	12 (1.00): drug_abuse gambling
	102 (0.25): action goal	
	43 (0.25): attention focus	
	16 (0.22): pain perception	
	54 (0.21): desire habit	
23	62 (0.83): reading language	24 (1.00): dyslexia specific_language_impairment
	72 (0.48): reading language	

The top five topics for each canonical variate exceeding a loading value of 0.2 are shown in the table.

## Discussion

It is clear that neuroimaging can provide important evidence regarding the functional organization of the brain, but one of the most fundamental questions in cognitive neuroscience has been whether it can provide any new insights into psychological function [17]–[19]. The results presented here demonstrate how large databases of neuroimaging data can provide new insights into the structure of psychological processes, by laying bare their relations within a similarity space defined by neural function. The present results highlight the importance of “discovery science” approaches that take advantage of modern statistical techniques to characterize large, high-dimensional datasets (cf. [20]). Just as the fields of molecular biology and genomics have been revolutionized by this approach [21], we propose that the hypothesis-generating approach supported by data mining tools can serve as a powerful complement to more standard hypothesis-testing approaches [22].

There is growing recognition that the diagnostic categories used in psychiatry are not reflective of sharp parallel biological distinctions; instead, a growing body of behavioral, genetic, and neuroimaging data suggest that these different disorders fall along a set of underlying continuous dimensions which likely relate to particular basic psychological processes [3], [4]. The results presented here are consistent with that viewpoint, and further show how endophenotypes for groups of disorders can be empirically discovered via data mining, even if those disorders were not the primary aims of the studies being mined. This approach would likely be even more powerful using databases that were focused on imaging data from studies of patients. In addition, this approach has the potential to characterize the genetic architecture of these disorders through mining of genetic association data; unfortunately, genetic terms are not sufficiently frequent in the Neurosynth database to support robust mapping of relationships to genes, but future analyses using enhanced databases has the potential to discover additional relations between neurocognitive components and genetic contributions.

The present work is limited by several features of the data that were used in the analyses. The first limitation arises from the fact that we rely upon the presence of particular terms in the text, rather than on manual annotation of the relevance of those terms. Thus, obvious issues such as polysemy (e.g., the multiple senses of the term “working memory”) and negation can be problematic, though these issues could potentially be addressed using more powerful natural language processing. A second limitation arises from the meta-analytic nature of the activation data used in the analyses, which are reconstructed from a very sparse representation of the original data. A third limitation is that the activation maps are associated only with complete documents, not with specific terms within the document, and this coarseness undoubtedly adds a significant amount of noise to the modeling results. These limitations necessitate caution in drawing strong conclusions from the results reported here. At the same time, the concordance of many of the results with previous analyses using different datasets and analysis approaches suggests that these limitations have not greatly undermined the power of the technique. We propose that the approach outlined here is likely to be most useful for inspiring novel hypotheses rather than for confirming existing hypotheses, which means that any such results will be just the first step in a research program that must also include hypothesis-driven experimentation.

Another potential limitation of the present work is that the fact that a number of the parameters in the analyses were set arbitrarily. While the dimensionality of the topic models was determined using an automated method, there remain parameter settings (such as smoothness of the word and topic distributions) that must be chosen arbitrarily (in our case, we chose them based on previously published results). The results of the topic model are quite robust; for example, we saw very similar results when performing the topic models on the original set of 4,393 papers from the earlier paper by Yarkoni et al. compared to the results from the corpus of 5,809 papers. It is also evident from Figure 5 that there is strong continuity in topics across different dimensionalities, with single topics at lower dimensionalities splitting into multiple finer-grained topics at higher dimensionalities. We have chosen model parameters that appear to give sensible results relative to prior findings, but the possibility remains that different parameterizations or analysis approaches could lead to different outcomes; future research will need to explore this question in more detail. We would also note that some of these limitations may be offset by the fact that the analyses presented here are almost fully automated, which removes many possible opportunities for research bias to affect the results.

The present work follows and extends other recent work that has aimed to mine the relations between mental function and brain function using coordinate-based meta-analyses. Smith et al. [23] analyzed the BrainMap database (which is similar to the database used here, but is created via manual annotation and thus has lower coverage but greater specificity and accuracy than the Neurosynth database). This work showed that independent components analysis applied to the meta-analytic data was able to identify networks very similar to those observed in resting-state fMRI time series, and that these could be related to specific aspects of psychological function via the annotations in the BrainMap database. Laird et al [24] extended this by showing that behavioral functions could be clustered together based on these meta-analytic maps. The present work further extends those previous studies by showing that the structure of the psychological domain can be identified in an unsupervised manner using topic modeling across both cognitive function and mental disorder domains, and that these can further be used to identify potential endophenotypes that share common neural patterns across these two domains. Visual examination of the ICA components presented in the Smith and Laird papers shows substantial overlap with the topic maps identified in the present study. In future work, we hope to directly compare the topic mapping results with the maps identified in those papers, to further characterize the utility of each approach.

In summary, we have shown how large neuroimaging and text databases can be used to identify novel relations between brain, mind, and mental disorders. The approach developed here has the potential to enable new discoveries about the neural and cognitive bases of neuropsychiatric disorders, and to provide empirically-driven functional characterizations of patterns of brain activation. The results also highlight the importance of the availability of large open datasets in cognitive neuroscience to enable discovery-based science as a complement to hypothesis-driven research.

## Materials and Methods

Code to implement all of the analyses reported here, along with all of the auxiliary files, are available at https://github.com/poldrack/LatentStructure.

### Data extraction

The full text from the Neurosynth corpus was used for the text mining analyses. The sources of these data as well as the process for automated extraction of activation coordinates are described in detail in [9].

### Peak image creation

Synthetic activation peak images were created from the extracted activation coordinates by placing a sphere (10 mm radius) at each activation location, at 3 mm resolution using the MNI305 template. Activations detected to be in Talairach space were first converted to MNI305 coordinates using the Lancaster transform [25].

### Topic modeling

We ran two topic modeling analyses using limited sets of terms to obtain focused topics in specific domains. In the first, we used 605 mental concept terms from the Cognitive Atlas database mentioned previously. In the second, we used a set of 55 terms describing mental disorders; these were obtained by taking the NIFSTD Dysfunction ontology and removing all terms not relevant to psychiatric disorders, and then adding a set of missing terms that described additional disorders listed in the DSM-IV. In each case, we processed the full text corpus and created restricted documents containing only terms that were present in the respective term list (along with synonyms, which were mapped back to the base term), and then performed topic modeling on those restricted documents. The median number of terms per document after filtering was 127 for cognitive terms and 3 for disease terms.

Topic modeling was performed using latent Dirichlet allocation [7] as implemented in the MALLET toolbox, version 2.0.6 [26], using 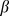 = 0.1 and 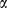 = 50/number of topics; these are the same values used by [8] in their analysis of PNAS abstracts. Optimization of topic-word hyperparameters was not used for the analyses reported here, as it tended to highly inflate the optimal number of topics.

For each dataset, the optimal number of topics was determined by performing a grid search across a range of dimensionality values (from 10 to 250 in steps of 10). Each document set was split into 8 random sets of documents, and 8 separate models were trained, in each case leaving out one subset of documents. The empirical likelihood of the left-out documents was then estimated using an importance sampling method as implemented in MALLET [10].

In order to identify the hierarchical relations between topics across different dimensionalities (as shown in Figure 5), the topic models from the first crossvalidation fold for each level (10, 50, 100, and 250 topics) were used; because 1/8 of the data were excluded as test data, these models were thus trained on a total of 5082 documents (using the same documents across all different dimensionalities). Hierarchical relations between levels were identified by computing the correlation between the document loading vectors for each lower-level topic and all higher-level topics, and then assigning the link according to the maximum correlation.

### Topic mapping

Topic maps were created separately for each topic by first computing a voxelwise chi-squared statistic for the association across documents between activation of the voxel (which is a binary feature due to the use of a spherical kernel) and the loading of that document on that topic (after thresholding the topic loading value p>0 and binarizing). This thresholding resulted in an mean number of documents per topic of 292 for the Cognitive Atlas analysis, and 177 for the neuropsychiatric disorders analysis. The voxelwise chi-squared p-value maps were then corrected for false discovery rate at q
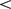
0.01 using the FSL fdr tool. Pearson correlation maps were also stored for use in the conjoint mapping and visualization.

### Disorder clustering

Disorders were clustered using hierarchical clustering (Ward's method) applied to the Euclidean distance matrix computed across voxels for the disorder-based topic maps (Pearson r values).

### Canonical correlation analysis

Canonical correlation analysis (CCA) was used to identify sets of mental function and disorder topics that were closely associated in neural activation space. In order to reduce the dimensionality of the data, the topic maps (Pearson r values) were first sampled from the original 3 mm space into 6 mm voxels. These datasets were then submitted to penalized canonical correlation analysis [15] using the PMA package in R (http://cran.r-project.org/web/packages/PMA/). The dimensionality of the decomposition was specified as 29 (i.e., the number of disorders); canonical variates were selected from this set for further analysis by thresholding the correlations between 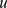 and 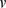 vectors at r
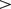
0.5. Penalty parameters were estimated using the permutation approach implemented in the CCA.permute function in the PMA package (best penalty = 0.63 for both dimensions). Loading vectors for the canonical variates (i.e., 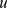 and 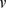 vectors) were constrained to be positive, in order to make the interpretation of the results clearer. The CCA analysis on document/topic mappings was performed identically, except that the document/topic vectors were used directly rather than mapping them into the neural activation space.

## Supporting Information

Table S1 Complete list of topics identified through application of latent Dirichlet allocation to the text corpus filtered for Cognitive Atlas terms. The top 5 words shown for each topic are those which had the highest loading for that topic across documents. The number of documents that loaded on each topic is also listed.
(PDF)

Table S2 Complete list of topics identified through application of latent Dirichlet allocation to the text corpus filtered for mental disorder terms. The top 5 words shown for each topic are those which had the highest loading for that topic across documents. The number of documents that loaded on each topic is also listed.
(PDF)
