# Supplementary material for: Discovering Relations Between Mind, Brain, and Mental Disorders Using Topic Mapping
Source: PLoS Comput Biol. 2012 Oct 11;8(10):e1002707. doi: 10.1371/journal.pcbi.1002707 (PMC3469446; doi:10.1371/journal.pcbi.1002707)
Supplement: Table S1 — Complete list of topics identified through application of latent Dirichlet allocation to the text corpus filtered for Cognitive Atlas terms. The top 5 words shown for each topic are those which had the highest loading for that topic across documents. The number of documents that loaded on each topic is also listed. (PDF) [file pcbi.1002707.s001.pdf]

Table S1. Complete list of topics identified through application of latent Dirichlet allocation to the text corpus filtered for Cognitive Atlas terms. The top 5 words shown for each topic are those which had the highest loading for that topic across documents. The number of documents that loaded on each topic is also listed.

Topic	Ndocs	Terms
46	948	search,visual_search,attention,conjunction_search,focus
74	769	auditory,perception,hearing,attention,listening
19	615	fixation,attention,movement,focus,goal
14	614	association,context,learning,attention,memory
88	608	focus,attention,association,context,knowledge
43	566	attention,focus,visual_attention,fixation,attentional_resources
86	519	decision,decision_making,choice,fixation,uncertainty
6	510	knowledge,semantic_knowledge,association,context,focus
20	497	action,movement,goal,context,perception
93	495	emotion,valence,arousal,attention,focus
90	494	inhibition,response_inhibition,attention,motor_inhibition,fixation
120	483	judgment,fixation,movement,decision,knowledge
31	481	perception,attention,visual_perception,focus,integration
102	476	action,goal,knowledge,encoding,reading
47	446	imagery,mental_imagery,visual_imagery,perception,auditory_imagery
89	444	action,movement,perception,integration,manipulation
61	442	memory,working_memory,maintenance,visual_working_memory,spatial_working_memory
105	437	emotion,sadness,context,happiness,attention
11	432	attention,sustained_attention,executive_control,memory,monitoring
116	430	action,action_selection,monitoring,focus,context
8	419	cognitive_control,monitoring,attention,performance_monitoring,goal
129	418	cognition,social_cognition,communication,theory_of_mind,context
128	409	movement,focus,attention,fixation,goal
107	401	language,language_processing,language_acquisition,language_production,learning
92	394	attention,spatial_attention,fixation,movement,selective_attention
106	391	movement,coordination,motor_control,feedback,planning
3	389	memory,episodic_memory,recall,learning,verbal_memory
111	386	memory,working_memory,attention,spatial_working_memory,cognitive_load
29	382	learning,error_signal,rule,goal,association_learning
118	379	integration,multisensory,crossmodal,unisensory,multisensory_integration
68	377	movement,feedback,search,attention,lying
70	377	memory,working_memory,efficiency,cognition,association
4	374	cognition,recognition,memory,social_cognition,object_recognition
62	367	reading,language,recognition,focus,fixation
44	356	risk,decision,utility,decision_making,monitoring
64	354	retrieval,memory,memory_retrieval,episodic_memory,encoding
13	349	language,comprehension,language_processing,meaning,semantic_processing
100	344	choice,decision,utility,reward,decision_making
124	341	feedback,learning,reward,focus,monitoring
39	340	valence,arousal,fixation,context,salience
82	336	meaning,subordinate,context,semantic_processing,language
112	335	context,social_context,integration,encoding,learning
21	334	interference,interference_resolution,cognitive_control,attention,proactive_interference
33	327	memory,retrieval,autobiographical_memory,memory_retrieval,episodic_memory
41	327	rule,rule_learning,context,learning,goal
9	321	executive_function,attention,memory,working_memory,inhibition
101	318	recognition,memory,retrieval,pattern_recognition,focus
69	317	concept,conceptualization,knowledge,meaning,indignation
109	312	manipulation,maintenance,object_manipulation,movement,focus
15	303	reward,anticipation,feedback,learning,movement
94	294	reward,decision,association,learning,choice
30	293	logic,task_set,task_switching,goal,fixation
52	290	retrieval,memory,memory_retrieval,retrieval_cue,encoding
73	289	arousal,attention,focus,excitement,context
58	289	emotion,facial_expression,recognition,emotion_recognition,fear
87	289	semantic_processing,semantic_information,retrieval,knowledge,meaning
7	286	encoding,memory,episodic_memory,retrieval,strategy
83	286	effort,cognitive_effort,attentional_effort,effortful_processing,efficiency
51	283	learning,sequence_learning,motor_learning,category_learning,motor_sequence_learning
79	282	familiarity,recognition,memory,context,encoding
113	282	encoding,memory,retrieval,recognition,focus
60	279	auditory,speech_production,perception,speech_perception,language
78	274	movement,motor_control,motor_execution,association,motor_program
36	274	memory,explicit_memory,declarative_memory,encoding,implicit_memory
77	274	mood,induction,emotion,sadness,happiness
76	272	planning,motor_planning,movement_planning,motor_execution,goal
97	271	awareness,consciousness,insight,attention,association
53	268	goal,context,goal_state,manipulation,knowledge
122	267	attention,selective_attention,divided_attention,attention_shift,inattention
63	264	monitoring,coordination,misattribution,context,effort
65	264	recall,humor,memory,retrieval,association
18	262	lying,deception,knowledge,executive_control,focus
55	262	recognition,object_recognition,word_recognition,object_identification,chunk
37	261	recognition,face_recognition,fixation,familiarity,attention
24	260	strategy,mental_arithmetic,focus,chunking,knowledge
125	256	skill,learning,procedural_learning,skill_learning,skill_acquisition
85	256	inference,knowledge,context,search,integration
67	255	maintenance,distraction,memory,working_memory,active_maintenance

34	254	perception,face_perception,fixation,apparent_motion,color_perception
28	254	salience,response_selection,manipulation,goal,choice
49	251	fear,emotion,attention,perception,context
98	248	stress,association,feedback,risk,mental_arithmetic
0	247	priming,decision,fixation,judgment,implicit_memory
26	243	comprehension,language,sentence_comprehension,language_comprehension,irony
2	241	adaptation,fixation,attention,priming,context
59	240	intelligence,morphology,fluid_intelligence,association,memory
50	235	listening,auditory,melody,perception,hearing
32	231	naming,retrieval,paraphasia,lexical_retrieval,phonemic_paraphasia
42	229	focus,cueing,cue_validity,attention,spatial_cueing
12	225	pain,perception,attention,inhibition,association
45	207	intention,prospective_memory,introspection,attention,intentionality
17	205	verbal_fluency,word_generation,articulation,language,word_repetition
10	205	rehearsal,memory,updating,working_memory,maintenance
38	204	categorization,prototype,fixation,category_learning,memory
96	196	context,context_memory,framing,focus,rule
117	196	anticipation,feedback,association,reward,context
127	191	memory,encoding,forgetting,emotional_memory,recognition
66	185	language,syntactic_processing,comprehension,syntax,sentence_processing
72	182	reading,language,skill,orthography,fixation
99	181	facial_expression,emotional_expression,perception,fixation,fear
119	179	competition,context,conflict_detection,manipulation,interference
40	178	fear,generalization,learning,association,awareness
115	171	rhythm,action,learning,encoding,imagery
5	169	word_frequency,decision,lexicon,reading,language
91	169	navigation,egocentric,movement,cognitive_map,memory
84	167	gaze,attention,perception,movement,context
23	165	pain,search,perception,distraction,cognitive_load
110	161	reasoning,deductive_reasoning,memory,knowledge,working_memory
104	157	feedback,auditory_feedback,auditory,movement,monitoring
75	156	retention,consolidation,memory,memory_consolidation,movement
126	153	automaticity,routine,learning,attention,interference
103	149	prosody,mental_rotation,intonation,perception,attention
80	149	expertise,attitude,prejudice,familiarity,stereotypes
123	142	stress,induction,memory,arousal,context
22	142	uncertainty,conversation,decision,monitoring,arousal
16	138	pain,perception,movement,manipulation,context
54	137	desire,habit,reward,association,decision
1	136	belief,theory_of_mind,reasoning,intention,fixation
81	128	hearing,auditory,perception,speech_processing,communication
25	127	memory,source_memory,encoding,recognition,decision
108	127	empathy,pain,theory_of_mind,awareness,facial_expression
114	124	acuity,grapheme,constancy,excitation,perception
57	120	semantic_memory,imageability,memory,knowledge,semantic_category
71	115	narrative,discourse,comprehension,memory,discourse_processing
56	114	expectancy,attention,arousal,focus,anticipation
27	89	analogy,reasoning,analogical_reasoning,integration,creative_thinking
121	68	regret,surprise,reasoning,arousal,learning
95	51	attachment,emotion,grief,association,sadness
35	44	hallucination,auditory,audition,language,association
48	41	metaphor,meaning,comprehension,irony,language
