# Supplementary material for: Discovering Relations Between Mind, Brain, and Mental Disorders Using Topic Mapping
Source: PLoS Comput Biol. 2012 Oct 11;8(10):e1002707. doi: 10.1371/journal.pcbi.1002707 (PMC3469446; doi:10.1371/journal.pcbi.1002707)
Supplement: Table S2 — Complete list of topics identified through application of latent Dirichlet allocation to the text corpus filtered for mental disorder terms. The top 5 words shown for each topic are those which had the highest loading for that topic across documents. The number of documents that loaded on each topic is also listed. (PDF) [file pcbi.1002707.s002.pdf]

Table S2. Complete list of topics identified through application of latent Dirichlet allocation to the text corpus filtered for mental disorder terms. The top 5 words shown for each topic are those which had the highest loading for that topic across documents. The number of documents that loaded on each topic is also listed.

**Topic**	**Ndocs**	**Terms**
12	245	drug_abuse,gambling,alcoholic_intoxication,antisocial_personality_disorder
11	242	aphasia
21	235	drug_abuse,alzheimers_disease,heroin_related_disorder,phobia
1	212	schizophrenia,drug_abuse
13	212	depressive_disorder,major_depressive_disorder,drug_abuse,bipolar_disorder,anxiety_disorder
14	199	drug_abuse,conduct_disorder,alcoholism,antisocial_personality_disorder,cannabis_related_disorder
16	197	schizophrenia
17	192	alcoholism,alexia
25	190	anxiety_disorder,panic_disorder,phobia,obsessive_compulsive_disorder,agoraphobia
6	189	schizophrenia,tic_disorder,mood_disorder
2	188	schizophrenia,psychotic_disorder,paranoid_disorder,drug_abuse,amphetamine_related_disorder
5	188	obsessive_compulsive_disorder,drug_abuse,tourettes_disorder,post_traumatic_stress_disorder,bipolar_disorder
4	187	psychotic_disorder,paranoid_disorder,schizophrenia,affective_psychotic_disorder,schizoid_personality_disorder
24	185	dyslexia,specific_language_impairment
20	181	attention_deficit_disorder,conduct_disorder,tourettes_disorder,adjustment_disorder
18	181	schizophrenia,psychotic_disorder,paranoid_disorder,cannabis_related_disorder
22	171	gambling,drug_abuse,impulse_control_disorder
15	170	attention_deficit_disorder
3	159	schizophrenia,paranoid_schizophrenia,obesity
23	156	autism,specific_language_impairment
7	154	bipolar_disorder,schizophrenia,mood_disorder,cyclothymic_disorder,alcoholism
28	151	borderline_personality_disorder,drug_abuse,post_traumatic_stress_disorder,alcoholism,panic_disorder
27	148	autism,asperger_syndrome,specific_language_impairment
19	147	autism,asperger_syndrome,capgras_syndrome
26	143	phobia,eating_disorder,agoraphobia,panic_disorder,impulse_control_disorder
0	142	mood_disorder,parkinsons_disease,huntingtons_disease,seasonal_affective_disorder
10	136	amnesia,alzheimers_disease,korsakoff_syndrome,wernicke_encephalopathy,trichotillomania
8	114	obesity,cocaine_related_disorder,drug_abuse,eating_disorder,alcoholism
9	112	schizophrenia,schizotypal_personality_disorder,paranoid_personality_disorder
